# Supplementary material for: Production of Metallic Alloy Nanowires and Particles Templated Using Tomato Mosaic Virus (ToMV)
Source: Nanomaterials (Basel). 2023 Oct 5;13(19):2705. doi: 10.3390/nano13192705 (PMC10574019; doi:10.3390/nano13192705)
Supplement: Supplementary file 1 [file nanomaterials-13-02705-s001.zip › nanomaterials-2625119-supplementary.pdf]

Supplementary Information:

Supplementary Figure S1.

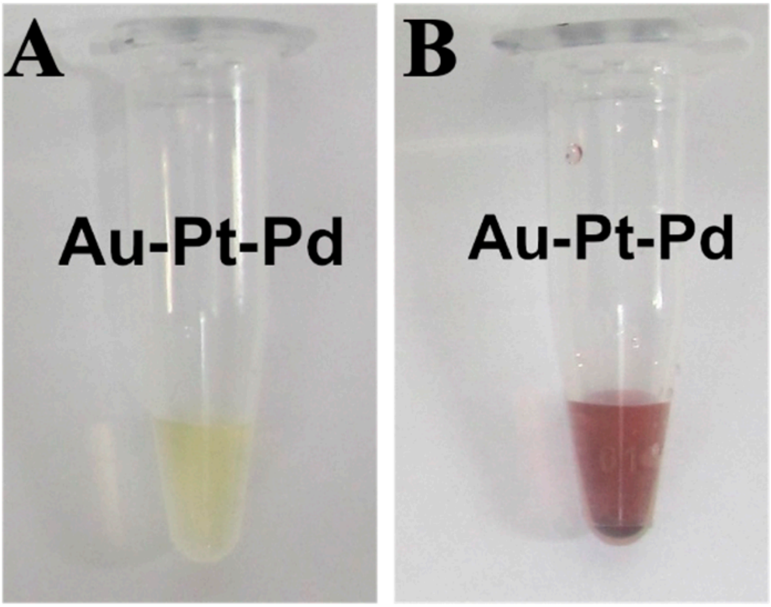

Supplementary Figure S2.

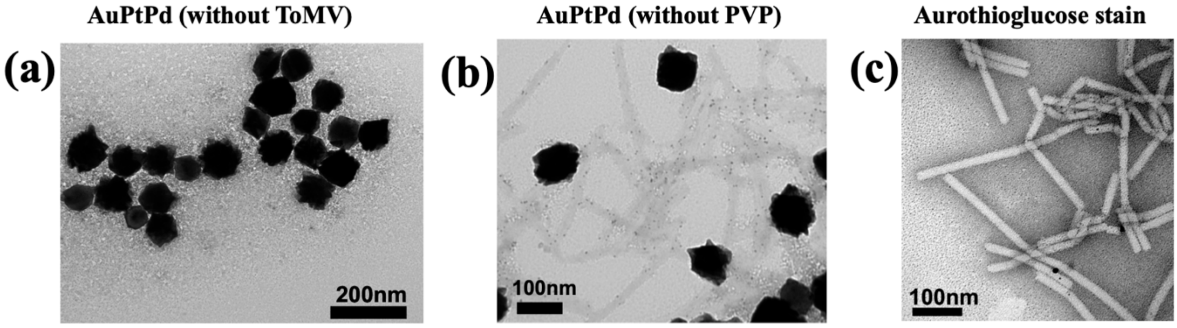

Supplementary Figure S3.

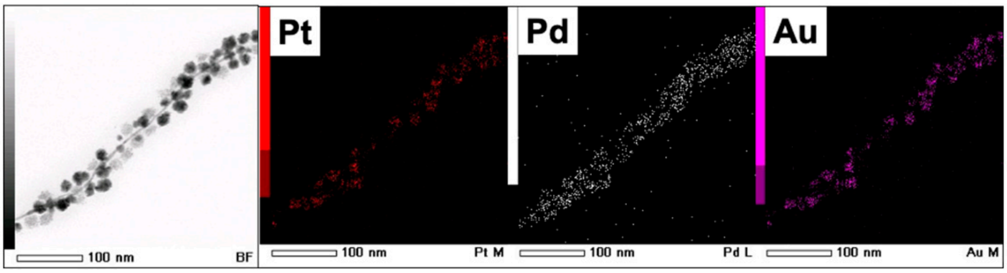

Supplementary Figure S4.

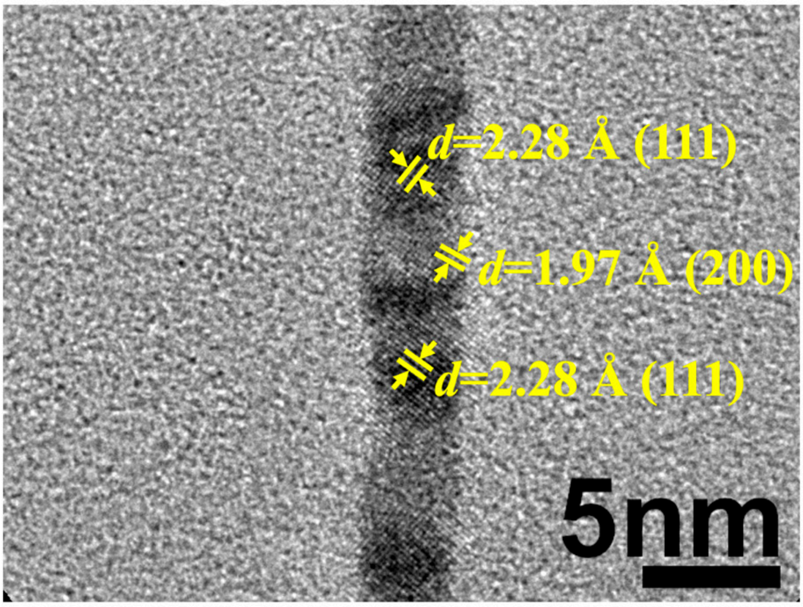

Supplementary Figure S5.

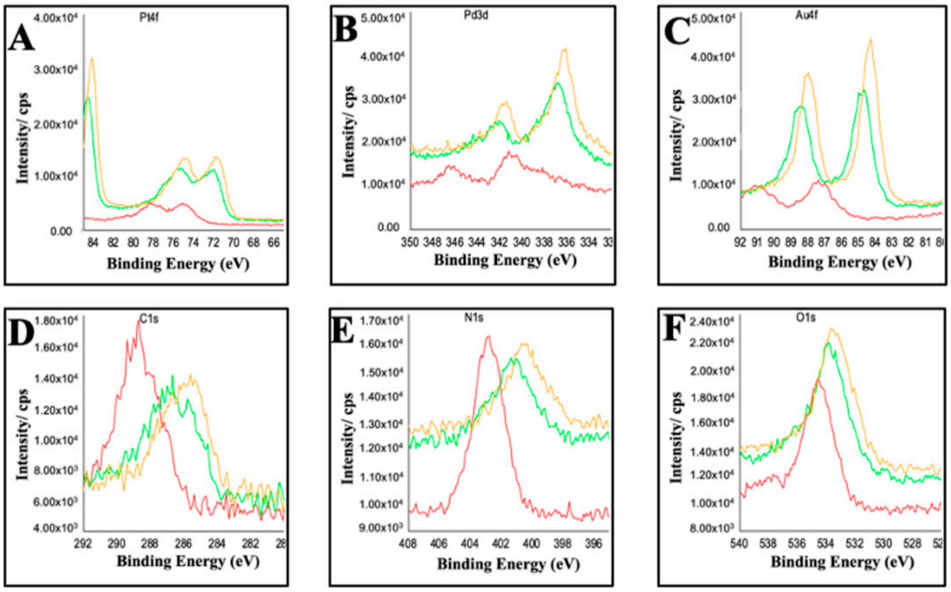

Supplementary Figure S6.

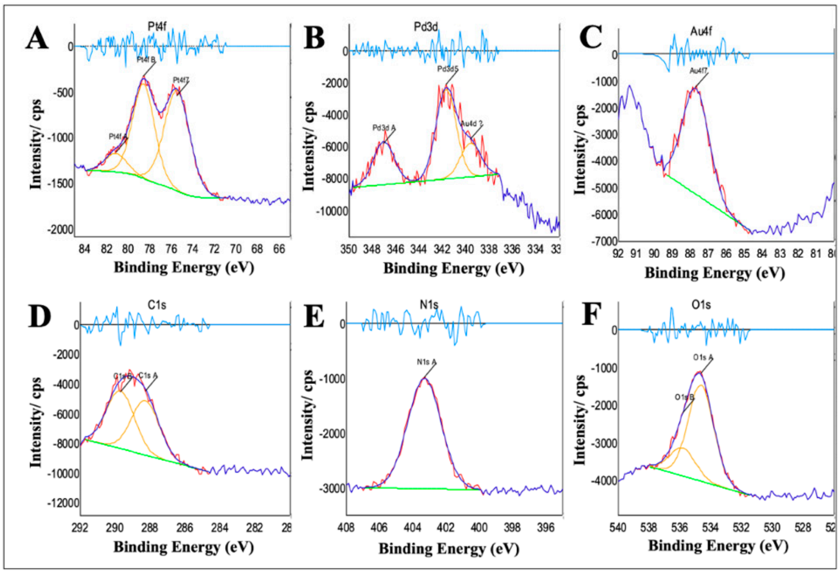

Supplementary Figure S7.

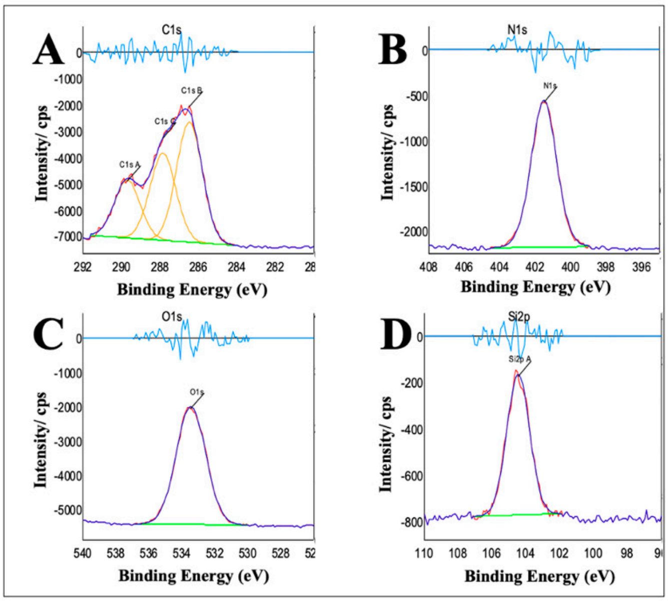

Supplementary Table S1:

Elements [a]	Peak	BE (eV)	Peak	BE (eV)
Au(0)	Au4f$_{7/2}$	84.0 eV	Au4f$_{5/2}$	88.0 eV
Pt(0)	Pt4f$_{7/2}$	71.2 eV	Pt4f$_{5/2}$	74.0 eV
Pd(0)	Pd3d$_{5/2}$	335.0 eV	Pd3d$_{3/2}$	340.0eV

[a] Handbook of X-Ray Photoelectron Spectroscopy. John F. Moulder, William F. Stickle, Peter E. Sobol, Kenneth D. Bomben. Edited by: Jill Chastain. Published by Perkin-Elmer Corporation, Physical Electronics Division, USA. 1992.

Supplementary Table S2:

	Peak position, BE (eV)			Peak position, BE (eV)			Peak position, BE (eV)	
Au	Au4f$_{7/2}$	Au4f$_{5/2}$	Pt	Pt4f$_{7/2}$	Pt4f$_{5/2}$	Pd	Pd3d$_{5/2}$	Pd3d$_{3/2}$
	84.2 eV (20s)	88.2 eV (20s)		72.0eV (20s)	75.0eV (20s)		336.2eV (20s)	341.8eV (20s)
	84.5 eV (10s)	88.4eV (10s)		72.2eV (10s)	75.2eV (10s)		336.8eV (10s)	342.2eV (10s)
	87.4 eV (0s)	91.0 eV (0s)		75.0eV (0s)	78.4eV (0s)		341.0eV (0s)	346.8eV 90s0
